# Psychometric properties of the Iranian interview-administered version of the World Health Organization's Quality of Life Questionnaire (WHOQOL-BREF): A population-based study

**DOI:** 10.1186/1472-6963-8-61

**Published:** 2008-03-21

**Authors:** Saharnaz Nedjat, Ali Montazeri, Kourosh Holakouie, Kazem Mohammad, Reza Majdzadeh

**Affiliations:** 1 School of Public Health, Tehran University of Medical Sciences, Tehran, Iran; 2 Iranian Institute for Health Sciences Research, Tehran, Iran

## Abstract

**Background:**

The objective of the current study was to translate and validate the Iranian version of the WHOQOL-BREF.

**Methods:**

A forward-backward translation procedure was followed to develop the Iranian version of the questionnaire. A stratified random sample of individuals aged 18 and over completed the questionnaire in Tehran, Iran. Psychometric properties of the instrument including reliability (internal consistency, and test-retest analysis), validity (known groups' comparison and convergent validity), and items' correlation with their hypothesized domains were assessed.

**Results:**

In all 1164 individuals entered into the study. The mean age of the participants was 36.6 (SD = 13.2) years, and the mean years of their formal education was 10.7 (SD = 4.4). In general the questionnaire received well and all domains met the minimum reliability standards (Cronbach's alpha and intra-class correlation > 0.7), except for social relationships (alpha = 0.55). Performing known groups' comparison analysis, the results indicated that the questionnaire discriminated well between subgroups of the study samples differing in their health status. Since the WHOQOL-BREF demonstrated statistically significant correlation with the Iranian version of the SF-36 as expected, the convergent validity of the questionnaire was found to be desirable. Correlation matrix also showed satisfactory results in all domains except for social relationships.

**Conclusion:**

This study has provided some preliminary evidence of the reliability and validity of the WHOQOL-BREF to be used in Iran, though further research is required to challenge the problems of reliability in one of the dimensions and the instrument's factor structure.

## Background

In recent years there has been increasing focus on measuring health beyond traditional indicators such as mortality and morbidity, and quality of life (QOL) has turned into an important outcome in clinical and interventional studies [1]. There is considerable agreement among experts that QOL is a multi-dimensional and subjective concept [2,3]. The World Health Organization Quality of Life Group defines quality of life as "individuals' perceptions of their position in life in the context of the culture and value systems in which they live and in relation to their goals, expectations, standards and concerns [4,5]."

There are many general instruments available to measure quality of life. The WHOQOL-BREF is one of the best-known instruments that has been developed for cross-cultural comparisons of quality of life and is available in more than 40 languages. It has been adopted in the United State of America, Netherlands, Poland, Bangladesh, Thailand, India, Australia, Japan, Croatia, Zimbabwe and many more other countries [4]. It is a shortened version of the WHOQOL-100 that looks at four quality of life profiles, using all available data from the field trial version of the WHOQOL-100 [4]. We selected this questionnaire because it is short and easy to use and to our best knowledge this is the first study that examines its psychometric properties in Iran employing the classical psychometric theory and methods. We thought this would allow to apply the questionnaire in both epidemiological and outcome studies and also could provide an opportunity for future research works to compare quality of life among Iranian population and people living in other communities.

## Methods

### The questionnaires

1. The WHOQOL-BREF is a 26-item instrument consisting of four domains: physical health (7 items), psychological health (6 items), social relationships (3 items), and environmental health (8 items); and two overall QOL and general health items. The physical health domain includes items on mobility, daily activities, functional capacity and energy, pain, and sleep. The psychological domain measures self-image, negative thoughts, positive attitudes, self-esteem, mentality, learning ability, memory and concentration, religion, and the mental status. The social relationships domain contains questions on personal relationships, social support, and sex life. The environmental health domain covers issues related to financial resources, safety, health and social services, living physical environment, opportunities to acquire new skills and knowledge, recreation, general environment (noise, air pollution, etc.), and transportation [5]. All scores are transformed to reflect 4 to 20 for each domain with higher scores corresponding to a better QOL. There is no overall score for the WHOQOL-BREF. Where an item is missing, the mean of other items in the domain can be substituted. Where more than two items are missing from the domain, the domain score should not be calculated, except for domain 3 in which more than one missing item is required to cancel the calculation. The questionnaires that have more than 20% missing items should be also excluded [5].

2. The SF-36 is a well known generic measure of health related quality of life consisting of eight subscales: physical functioning, social functioning, vitality, role limitations due to emotional problems, bodily pain, role limitations due to physical problems, mental health, and general health. The psychometric properties of the Iranian version of the SF-36 (interview-administered) are well documented [6].

### Translation

Two experienced Iranian health professionals, bilingual in Persian (the Iranian language) and English, independently translated the English version of the WHOQOL-BREF into Persian. Then, a member of the research team (AM) produced the consolidated forward version. In case there were differences between two translated versions, the problem was resolved through discussion with the translators to yield a provisional forward translation. If there was fundamental disagreement a third independent translator was invited to judge. Two independent translators, whose mother tongue was English and who had no previous knowledge of the questionnaire, then translated the instrument back to English. Discrepancies were discussed in the bilingual expert panel and in case there were differences that could not be resolved through discussion, agreement was reached by revision of the forward translation. Finally the provisional version of the Iranian questionnaire was provided and pilot tested to assess the feasibility and clarity of the items and response categories [4,5,7]. A convenient sample of 56 individuals participated in the pilot study. They were asked to respond to a short questionnaire to indicate items difficult to understand, confusing or offensive.

### Study population and data collection

The WHOQOL-BREF was administered to a random sample of individuals aged 18 and above (with no upper age limitation) living in all 22 districts of Tehran, selected through a stratified multi-stage area sampling. The Iranian Students' Polling Agency (ISPA) carried out the sample size calculation and recruitment. To avoid selection bias related to illiterate participants and also to reduce number of missing responses, we decided to use interviews instead of self-completion mode for data collection. All participants were interviewed individually in their homes by a team of trained interviewers. In this random sample there were healthy individuals (who stated to be free of a chronic medical condition and not receiving any therapeutic interventions), and ill participants (who reported to have one or more medical conditions receiving some form of medical care). These data were used for internal consistency and known groups' comparison analyses. Every tenth respondent in the healthy sample was asked to also respond to the SF-36 in the interview, and in total 95 participants were interviewed with the SF-36 to assess convergent validity. They also completed the WHOQOL-BREF and the SF-36 two weeks later, to assess test-retest reliability. The same individual carried out both test and retest interviews. The ethics committee of Tehran University of Medical Sciences approved the research and all participants gave their verbal informed consents.

### Statistical analysis

Psychometric properties of the WHOQOL-BERF were assessed using different statistical tests as follows.

#### Internal consistency

The internal consistency for each domain was estimated using Cronbach's alpha. Values equal to or greater than 0.70 were considered satisfactory [8-10]. In addition, descriptive statistics, floor and ceiling effects were also reported.

#### Test-retest

Reliability of the WHOQOL-BREF was assessed in a random sample of healthy individuals performing intraclass correlation analysis (ICC – the two-way random model) using the SPSS11.5 software. The same interviewer carried out the test and retest interviews. The ICC is an estimate of the fraction of the total measurement variability due to variation among individuals [2,8,10]. We expected that the ICC for each WHOQOL-BREF domain, the overall QOL and the general health item to exceed 0.7.

#### Convergent and divergent validity

A moderate correlation (0.45 < r < 0.70) between the physical domain of the WHOQOL-BREF and physical functioning, physical pain, physical role and general health subscales of the SF-36 was expected. Likewise, it was expected that the psychological and social relationships of the WHOQOL-BREF be moderately correlated (0.45 < r < 0.70) with the social functioning, emotional role, vitality and mental health subscales of the SF-36. The other domains were predicted to have weaker correlations (r ≤ 0.45) that demonstrate divergent validity [8]. It is believed that in the convergent validity we should not expect high correlation between two instruments; instead a moderate correlation would be preferable. However if two instruments show high correlation one should have the brevity or feasibility advantage to another as a reason for validation [10].

#### Known groups' comparison

The ability of the WHOQOL-BREF to discriminate between healthy and ill groups was tested by performing analysis of covariance (ANCOVA) that controls the effects of confounders such as age, sex, educational level, marital status, and the proxy for economic status (home size in meter^2^/number of households) [11]. A factor was recognized as a confounder if it affected the domain scores independently and if it showed significant effect in ANCOVA. We expected that the healthy group would have significantly higher scores in all domains compared to the ill group.

#### Correlation matrix of the WHOQOL-BREF

The correlation between 26 items and 4 domains was assessed and correction for overlap was also applied. Overall, we expected items to correlate more strongly with the domains to which they were originally assigned (r equal to or greater than 0.40).

## Results

### Pilot testing

There were no problems regarding either the items or response categories. However, a few changes were made on the basis of the pre-test results. Since in Iran asking people about their sexual life is sensitive, the question regarding sexual life was changed to 'sexual relationship' for married, divorced and separated people and 'relationship with opposite sex' for never married respondents.

The time required for completion of the WHOQOL-BREF ranged from 3 to 15 minutes with a median of 6 minutes. The reason for this wide range in time was that the samples in the pilot study were chosen from a wide range of educational levels. Also the subjects had different health status, so that for very ill and less-educated participants, the time needed to fill in the questionnaire was more than healthy and better-educated participants.

### The study sample

In all, 1210 individuals were interviewed. Forty-six questionnaires had more than 20% missing data and thus were excluded from the study. The analysis was restricted for the remaining 1164 respondents. Of these 906 people were healthy and 258 participants indicated having one or more medical conditions. Overall, 209 households refused to participate in the study. The main reason for refusal was a general dislike. There was no difference between respondents (n = 1164) and non-respondents (n = 209) in age [mean (SD): 36.6 (13.2) and 35.1 (16.0), respectively], sex (52.1% and 52.0% female, respectively), and education [mean year (SD): 10.7 (4.4) and 10.2 (4.5), respectively].

The characteristics of the respondents are shown in Table 1. The mean age of the study groups (healthy and ill) were different, and educational level in the group having medical conditions was slightly lower than the other group. The major chronic conditions stated by the participants included musculoskeletal pains (40%), cardiovascular diseases (28%), psychological disorders (10%) and endocrinological diseases such as diabetes and thyroid dysfunction (15%). Because of differences in age, gender and level of education between the study samples, we controlled the effect of these variables.

**Table 1:** Characteristics of the study participants

	**All (n = 1164)**	**Healthy individuals (n = 906)**	**Ill individuals (n = 258)**
**Age (years)**			
Mean (SD)	36.6 (13.2)	34.3 (12.4)	44.9 (12.5)
**Education (years)**			
Mean (SD)	10. 7 (4.4)	11.3 (4.0)	8.9 (5.1)
**Sex (%)**			
Male	47.9	50.9	37.6
Female	52.1	49.1	62.4
**Marital status (%)**			
Single	27.5	32.5	12
Married	67.8	63.7	81.4
Widowed/separated	4.6	3.8	6.6

### Descriptive statistics and internal consistency

Table 2 illustrates the missing, floor and ceiling effects for each item. The percentage of respondents scoring at the lowest level (i.e., floor effect) ranged from 1 to 22.3 while the percentage of respondents scoring at the highest level (i.e., ceiling effect) ranged from 1.8 to 45.3.

**Table 2:** Response pattern and missing items for each item (n = 1164)

**Items (item numbers)**	**Missing (%)**	**Mean score**	**SD**	**Floor (%)**	**Ceiling (%)**
Overall QoL (1)	0.2	3.4	0.86	3.0	6.9
Overall Health (2)	00	3.6	0.97	2.5	15.4
Pain (3)	00	3.9	1.2	4.6	45.3
Dependence of medical aids (4)	0.3	3.9	1.1	3.0	41.7
Positive feeling (5)	0.1	3.1	1.1	9.5	9.1
Personal belief (6)	0.3	3.4	1.1	6.4	14.9
Concentration (7)	0.3	3.3	0.99	4.0	10.5
Security (8)	0.3	3.4	1.1	5.8	14.2
Physical environment (9)	0.3	3.1	1.1	10.5	11.1
Energy (10)	0.1	3.4	0.88	1.9	6.6
Bodily image (11)	0.2	3.6	0.86	2.6	11.0
Financial support (12)	00	2.9	0.88	5.2	2.7
Accessibility of information (13)	0.7	3.1	0.85	2.4	3.1
Leisure activity (14)	0.3	2.4	1.00	22.3	1.8
Mobility (15)	0.3	3.5	0.92	2.3	9.8
Sleep and rest (16)	00	3.4	1.1	5.5	12.5
Activities of daily living (17)	0.3	3.5	0.86	1.9	7.1
Work capacity (18)	0.5	3.4	0.91	2.3	7.7
Self-esteem (19)	0.2	3.5	0.95	2.7	14.6
Personal relationship (20)	0.1	3.7	0.83	1.00	13.7
Sexual activity (21)	2/5	3.5	0.85	2.7	6.4
Social support (22)	00	3.2	1.00	6.7	8.8
Home environment (23)	0.3	3.3	1.00	5.8	9.7
Health care (24)	0.3	3.3	1.00	7.3	7.2
Transport (25)	0.1	3.2	1.00	8.1	5.2
Negative feeling (26)	0.1	3.2	1.2	8.2	19.2

Table 3 presents the internal consistency for the four WHOQOL-BREF domains. All domains except social relationships met or exceeded the 0.7 level recommended as an acceptable internal consistency. The table also shows the mean scores for all participants.

**Table 3:** Descriptive and reliability statistics for the WHOQOL-BREF

	**Descriptive statistics**		**Alpha coefficients**	
**Domains**	Number of items	All (n = 1164) Mean score (SD)*	Healthy individuals (n = 906)	Ill individuals (n = 258)
**Physical health**	7	14.3 (2.6)	0.70	0.74
**Psychological health**	6	13.4 (2.6)	0.73	0.70
**Social relationships**	3	13.9 (2.6)	0.55	0.61
**Environmental health**	8	12.3 (2.4)	0.84	0.72

* The higher score represents a better condition (scores range from 4 to 20).

### Test-retest

In test-retest analysis, the ICCs for the four domains were within the range of acceptable values (physical health = 0.77; psychological health = 0.77; social relationships = 0.75; and Environmental health = 0.84). The ICC for the overall QOL and the general health items was 0.69. The ICC for each item ranged from 0.51 to 0.74 with a median equal to 0.61.

### Convergent and divergent validity

Table 4 presents the convergent validity results. Most correlations were in the expected direction. However, divergent validity was not consistently achieved. For example, the physical domain of the WHOQOL-BREF had a correlation of more than 0.5 with the SF-36 social functioning, mental health and emotional role subscales.

**Table 4:** The Pearson correlations between the WHOQOL-BREF and the SF-36* (n = 95)

	**Physical health**	**Psychological health**	**Social relationships**	**Environmental health**
**Bodily pain**	0.57	0.19	0.10	0.31
**General health**	0.64	0.43	0.21	0.42
**Vitality**	0.65	0.58	0.29	0.47
**Physical functioning**	0.47	0.38	0.21	0.33
**Role physical**	0.55	0.32	0.18	0.33
**Mental health**	0.50	0.56	0.27	0.38
**Role emotional**	0.52	0.52	0.08	0.46
**Social functioning**	0.67	0.49	0.33	0.47

*: Weak correlation (r ≤ 0.45); moderate correlation (0.45 < r < 0.70); strong correlation (r ≥ 70).

### Known groups' comparison

The adjusted means for the four WHOQOL-BREF domains are presented in Table 5. Scores in all domains were significantly different in these groups. Overall, the differences were as expected, such that the healthy groups' scores were higher than the ill. In the physical health domain, the confounders were educational level and age; in the psychological domain, only educational level was recognized as a confounder; and in social relationships, marital status, age and sex. Sex and economic status were the confounders of the environmental domain score.

**Table 5:** The known groups' comparison (controlled for confounders)

	**Healthy individuals **(n = 906)	**Ill individuals **(n = 258)	**P value**
	
	Mean* (SD)	Mean* (SD)	
**Physical health**	14.7 (2.3)	12.9 (2.7)	< 0.001
**Psychological health**	13.7 (2.5)	12.5 (2.6)	< 0.001
**Social relationships**	14.1 (2.4)	13.4 (2.9)	0.001
**Environmental health**	12.7 (2.4)	11.5 (2.4)	< 0.001

* Adjusted mean. The higher score represents a better condition (scores range from 4 to 20).

### WHOQOL-BREF correlation matrix

Table 6 presents correlations between the WHOQOL-BREF questions and domains. As expected, most questions showed the highest correlations with domains to which they were originally assigned. However, 4 questions did not correlate very well with their related domains: 2 questions of the social relationships (sexual activity and personal relationships) showed the highest correlation with psychological domain, and 1 question (social support) of this domain showed the highest correlation with the environmental health domain. The question of bodily image of the psychological health domain demonstrated highest correlation with the physical health domain.

**Table 6:** Item-scale correlation matrix for the four WHOQOL-BREF measures* (n = 1164)

	**Physical health**	**Psychological health**	**Social relationships**	**Environmental health**
**Physical health (item number)**				
Pain (3)	**0.55**	0.34	0.18	0.25
Dependence of medical aids (4)	**0.52**	0.29	0.18	0.25
Energy (10)	**0.58**	0.47	0.26	0.37
Mobility (15)	**0.53**	0.40	0.24	0.28
Sleep and rest (16)	**0.34**	0.30	0.23	0.26
Activities of daily living (17)	**0.62**	0.48	0.32	0.36
Work capacity (18)	**0.48**	0.46	0.30	0.32
**Psychological health (item number)**				
Positive feeling (5)	0.40	**0.56**	0.37	0.49
Personal belief (6)	0.31	**0.52**	0.33	0.41
Concentration (7)	0.33	**0.44**	0.28	0.34
Bodily image (11)	0.35	**0.27**	0.16	0.18
Self-esteem (19)	0.41	**0.43**	0.38	0.30
Negative feeling (26)	0.39	**0.41**	0.29	0.34
Social relationships (number of items)				
Personal relationship (20)	0.28	0.39	**0.36**	0.25
Sexual activity (21)	0.25	0.32	**0.26**	0.27
Social support (22)	0.17	0.27	**0.24**	0.35
**Environmental health (item number)**				
Security (8)	0.27	0.36	0.27	**0.46**
Physical environment (9)	0.12	0.25	0.23	**0.44**
Financial support (12)	0.32	0.45	0.29	**0.48**
Accessibility of information (13)	0.25	0.31	0.24	**0.37**
Leisure activity (14)	0.34	0.42	0.30	**0.44**
Home environment (23)	0.19	0.27	0.31	**0.45**
Health care (24)	0.28	0.26	0.25	**0.48**
Transport (25)	0.33	0.31	0.28	**0.43**

* Pearson correlation (r) equal to or greater than 0.40 was considered satisfactory.

## Discussion

This study aimed to validate the Iranian version of the WHOQOL-BERF. The study recruited a relatively large representative sample form the general population. In general the results were promising although there were some problems and difficulties. The WHOQOL-BREF was basically designed to be a self-administered questionnaire but to prevent the selection bias due to illiterate participants and to reduce missing data we used interviews. Therefore one might argue that the self-administered version of the questionnaire will require further psychometric testing. In addition, it seems that reliability of the social relationships domain and its items' correlations need more investigations.

According to Table 6, 83% (20/24) of the WHOQOL-BREF questions showed maximum correlation with their original domains in the expected directions, but the 3 questions of social relationships and question of psychological health demonstrated maximum correlation with the other domains.

Since the intra-class correlation coefficient in all domains was more than 0.70, the questionnaire's reproducibility was satisfactory [12]. Internal consistency was measured using the Cronbach's alpha coefficient. The study findings indicated satisfactory alpha coefficients in all domains except for the social relationships. This domain showed similar results in validation studies in different countries, and also the WHOQOL-BREF field trial reported a Cronbach's alpha less than 0.7 in this domain [13-15]. This can be attributed to the small number of questions (3 items) in this domain. In addition, this domain does not appear very homogenous at least in the Iranian culture, since it inquires about sexual life and social supports that are relatively different concepts in Iranian culture. We should consider revising these items to yield a stronger result in the future studies. Although the social relationships domain did not have the expected internal consistency, the questionnaires with similar or poorer consistency were applied as valid tools [13-15]. However some questions can be added to the end of the questionnaire in a separate sheet in the future after necessary evaluations are done. We are planning to do so and this will be reported in due course.

The correlations between the WHOQOL-BREF and the SF-36 were as expected in most items. A correlation higher than 0.45 was found between the psychological domain of the WHOQOL-BREF and physical role and social functioning subscales from the SF-36 that were more than the expected size. This can be attributed to overlapping domains in the WHOQOL-BREF [8]. Moreover, the SF-36 validation also showed strong correlations between the physical and mental domains in the Iranian population [6]. On the other hand, the SF-36 questions are more objective in comparison to the completely subjective questions of the WHOQOL-BREF. However, evidence suggests that these tools do not measure exactly the same constructs. The evidence suggests that WHOQOL-BREF is more sensitive to demographic characteristics of participants [16,17].

Regarding the known groups comparison, there were significant differences between the two ill and healthy groups after controlling for confounders. We concluded that the questionnaire has acceptable discriminative validity. As it was demonstrated in Table 5, the differences between healthy and ill groups in all domains were significant.

## Conclusion

The WHOQOL-BREF questionnaire is a brief and useful instrument to measure quality of life. This study has provided some preliminary evidence of the reliability and validity of the WHOQOL-BREF for use in Iran, though further research is required to challenge problems of reliability in one dimension and the instrument's factor structure.

## Competing interests

The author(s) declare that they have no competing interests.

## Authors' contributions

Saharnaz Nedjat and Ali Montazeri participated in the design, and statistical analysis and wrote the manuscript. Kourosh Holakoui participated in the design and conducting the study. Kazem Mohammad helped to analyz data and Reza Majdzadeh participated in the study design and helped in writing. All authors read and approved the final manuscript.

## Pre-publication history

The pre-publication history for this paper can be accessed here:


